# Solitary Hepatic Metastasis of Thymoma: A Case Report

**DOI:** 10.70352/scrj.cr.25-0400

**Published:** 2026-02-13

**Authors:** Hiroshi Sakai, Yoshiaki Tominaga, Kazuya Yoshida, Ian Fukudome, Yoshiki Mizukami, Tomofumi Watanabe, Kenji Sano, Manabu Hiraguri

**Affiliations:** 1 Department of Surgery, Iida Municipal Hospital, Iida, Nagano, Japan; 2 Department of Radiology, Iida Municipal Hospital, Iida, Nagano, Japan; 3 Department of Pathology, Iida Municipal Hospital, Iida, Nagano, Japan

**Keywords:** thymoma, recurrence, hepatic metastasis, hepatectomy

## Abstract

**INTRODUCTION:**

Hepatic recurrence of thymomas is uncommon and its surgical treatment has rarely been reported. We report the case of a patient who underwent surgical resection for a solitary liver metastasis of a thymoma.

**CASE PRESENTATION:**

A 61-year-old female presented with an abnormal shadow on chest radiography performed during a medical checkup. CT revealed an 85-mm mass in the anterior mediastinum, adjacent to the pericardium and the right upper and right middle lobes of the lung. The patient underwent total thymectomy, wedge resection of the upper and middle lobes of the right lung, and partial resection of the pericardium. Histopathology revealed a type B3 thymoma, classified as stage II according to the Masaoka staging system. An abdominal CT performed 18 months after the primary surgery revealed a 34-mm solid mass in the 6th liver segment. Wedge resection of the hepatic lesion was performed. Pathological examination revealed liver metastasis of a type B3 thymoma. No recurrence was observed during follow-up.

**CONCLUSIONS:**

The paucity of data on the surgical treatment of liver metastases from thymomas hinders the establishment of clear evidence-based guidelines for determining the indications for surgical intervention. Surgery appears to be the most appropriate treatment for solitary liver metastasis; however, the most efficacious treatment remains to be elucidated. It is necessary to accumulate more cases of patients who have undergone hepatectomy for liver metastasis of thymoma.

## Abbreviations

CD
: cluster of differentiation
FDG-PET/CT
: fluorodeoxyglucose-PET/CT
ITMIG
: International Thymic Malignancy Interest Group
SUVmax
: maximum standardized uptake value

## INTRODUCTION

Thymomas are rare but the most frequent tumors arising from the anterior mediastinum.^1,2)^ Significantly better survival has been reported in patients who underwent complete resection. Therefore, surgical resection is the mainstay of treatment, when technically feasible.^1–3)^ Although generally indolent, thymomas can metastasize to the pleura, pericardium, or distant sites.^1)^ Recurrence rates are reported to be 10%–15% in resected thymoma cases.^2,3)^ Although the most common site of metastasis and recurrence is the pleura, followed by the lungs, thymomas can also cause liver metastasis. However, only few resected cases have been reported in the literature. Here, we report the case of a patient who underwent surgical resection for a solitary liver metastasis of a thymoma.

## CASE PRESENTATION

**Fig. 1:**
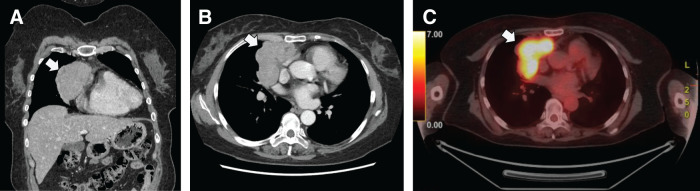
(**A**, **B**) Chest CT findings. An 85-mm mass in the anterior mediastinum with a lobulated contour and heterogeneous internal enhancement. The tumor was adjacent to the pericardium and the right upper and right middle lung lobes. (**C**) 18F-fluorodoeoxyglucose PET/CT showed increased 18F-fluorodoeoxyglucose uptake (maximum standardized uptake value [SUVmax], 12.8) at the tumor site.

A 61-year-old female presented with an abnormal shadow on chest radiography performed during a medical checkup. Chest CT revealed an 85-mm mass in the anterior mediastinum with a lobulated contour and heterogeneous internal enhancement. The tumor was adjacent to the pericardium and the right upper and right middle lung lobes (**Fig. 1A** and **1B**). ^18^F-FDG-PET/CT showed increased ^18^F-fluorodeoxyglucose uptake (SUVmax, 12.8) at the tumor site (**Fig. 1C**). No symptoms suggestive of myasthenia gravis were present. The preoperative anti-acetylcholine receptor antibody test results were negative. The tumor was suspected to be a thymoma without myasthenia gravis, with infiltration into the pericardium and the upper and middle lobes of the right lung, clinically classified as Masaoka stage III.

**Fig. 2:**
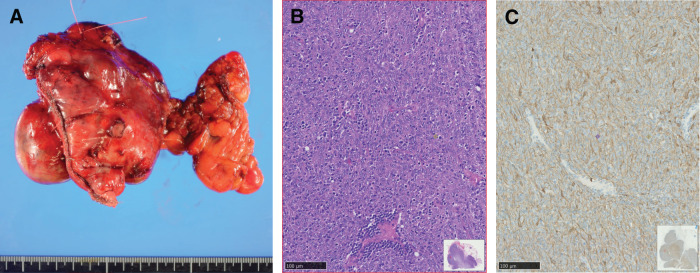
(**A**) Macroscopic image of the excised thymic tumor. (**B**) Microscopic finding from the resected tumor specimen. Hematoxylin and eosin staining of the tumor. (**C**) Immunohistochemical studies revealed that the tumor cells were positive for cytokeratin AE1/AE3.

Surgical resection was performed through median sternotomy. The tumor was located on the right portion of the thymus. It showed strong adhesion to the surrounding tissues of the thymus, especially the pericardium, and the upper and middle lobes of the right lung. We performed a total thymectomy, partial resection of the pericardium, and wedge resection of the upper and middle lobes of the right lung (**Fig. 2A**). Microscopically, the tumor had not invaded the lung parenchyma or pericardium, though it had invaded the capsule. A complete microscopic resection was achieved. Hematoxylin and eosin (H&E) staining of the resected specimen revealed that the tumor showed thymic epithelium-predominant proliferation associated with non-neoplastic immature T cells. The tumor cells showed mildly or moderately atypical polygonal nuclei with a sheet-like, solid growth pattern (**Fig. 2B**). Immunohistochemical studies revealed that the tumor cells were positive for cytokeratin AE1/AE3 (**Fig. 2C**) and negative for CD 5 and c-kit; the lymphocytes were positive for terminal deoxynucleotidyl transferase and CD 5. Histological findings were consistent with those of type B3 thymomas, according to the World Health Organization (WHO) classification. The tumor was pathologically diagnosed as type B3 thymoma (Masaoka stage II). The patient was discharged in a good condition after 8 days.

**Fig. 3:**
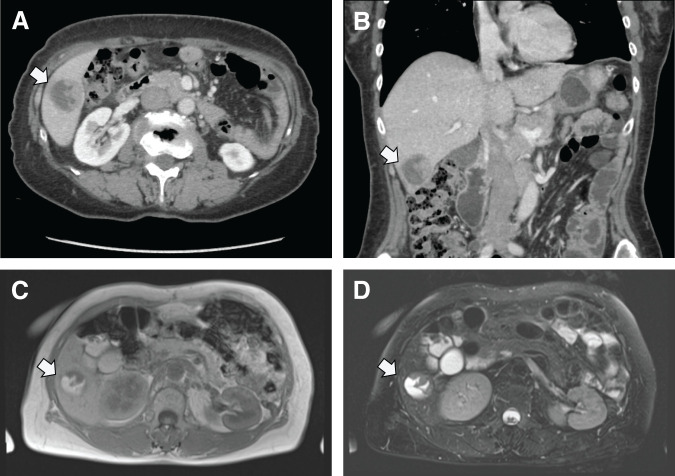
(**A**, **B**) Abdominal CT findings of a 3.4-cm solid mass in the 6th liver segment. The tumor was well defined and rounded, with heterogeneous internal enhancement after contrast injection. (**C**, **D**) On MRI, the tumor exhibited signal characteristics similar muscle on T1-weighted images and a higher signal than muscle on T2-weighted images. In addition, it showed fluid characteristics of cysts with high water content.

During regular follow-up 18 months after the primary surgery, an abdominal CT showed a 3.4 cm solid mass in the 6th liver segment. The tumor was well defined and rounded, with heterogeneous internal enhancement after contrast injection (**Fig. 3A** and **3B**). This finding suggested hemorrhage or necrosis within the tumor. On MRI, the tumor exhibited signal characteristics similar muscle on T1-weighted images and a higher signal than muscle on T2-weighted images. In addition, it showed fluid characteristics of cysts with high water content (**Fig. 3C** and **3D**). This tumor heterogeneity was similar to that observed in the CT studies. The tumor was suspected to be a liver metastasis of the thymoma.

**Fig. 4:**
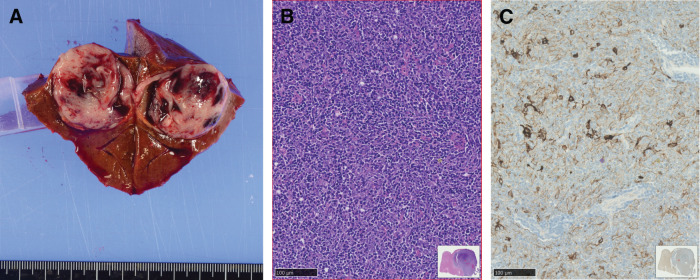
(**A**) Gross view of a cross section of the liver tumor. The lesion, measuring 3.5 cm, was seen to consist of a grayish-white solid tumor with a massive intratumor hematoma. (**B**) Hematoxylin-eosin staining of the liver tumor. (**C**) Immunohistochemical studies revealed that the tumor cells were positive for cytokeratin AE1/AE3.

The patient underwent a wedge resection of the hepatic lesion (**Fig. 4A**). Pathological examination confirmed it to be a liver metastasis of a type B3 thymoma. The histopathological findings were consistent with those of the primary tumor (**Fig. 4B** and **4C**). The patient was discharged in a good condition after 10 days.

The patient remained alive and asymptomatic 16 months after hepatectomy. However, CT imaging of the chest 9 months after hepatectomy revealed a 7-mm small nodular lesion in the right lower lobe of the lung that had not been detected on CT prior to liver resection. No significant changes were observed in the small nodule on a subsequent CT scan 16 months following the hepatectomy, and the nodule remained under meticulous surveillance.

## DISCUSSION

Thymomas represent 50% of anterior mediastinal masses in patients >50 years old, and 70% if substernal goiters are excluded.^1)^ The overall incidence of thymomas in the United States is 0.13 per 100000 person-years.^4)^ In 1978, Bergh et al.^5)^ recognized that all thymic tumors can exhibit malignant behavior and should be treated as malignant diseases. Long-term survival and recurrence-free rates are excellent following complete tumor resection. Therefore, surgical resection is the mainstay of treatment, and achieving complete resection is the most important prognostic factor, necessitating every effort at the time of surgery.^1)^ Data from the Japanese Association for Thoracic Surgery indicate that 2174 thymomas were resected in 2021.^6)^

Despite their generally indolent behavior, recurrences are common. Weis et al.^7)^ reported that 434 (10.28%) of 4221 thymomas from the ITMIG database experienced recurrence. The 5-year recurrence rates for types A, AB, B1, B2, and B3 were 4%, 2%, 8%, 13%, and 14%, respectively. The “type A, AB, B1–B3 thymoma” nomenclature was originally introduced by Dr. Juan Rosai for the major thymoma types in the second edition of the WHO classification in 1999. This nomenclature has since been globally accepted and maintained in the fifth edition of the WHO classification of thymic epithelial tumors.^8)^ According to the ITMIG, recurrence is classified according to the recurrence site as local (mediastinum), regional (pleural cavity), or distant (i.e., hematogenous spreading).^9)^ Recurrence was local or regional in most cases, whereas distant recurrence occurred in 2%–27% of cases.^10)^ Among distant metastases, the lungs are the most involved organ, and abdominal recurrences are rare. Liver metastases are uncommon; however, among extra thoracic metastatic sites, the liver is the second most common.^11)^ Previous reports indicate that liver metastasis is rare, occurring in 1.2%–21.4% of patients with recurrence (**Table 1**).^2,11–17)^ Although Khandelwal et al.^16)^ reported higher rates of liver metastasis, their data included all sites of involvement through the course, rather than the sites at the time of the first episode of metastasis or progression.

**Table 1:** Reported cases of thymoma recurrence and liver metastases

Authors	Year		Number of patients		Liver metastasis/ Thymoma (%)	Liver metastasis/ Recurrence (%)
		Thymoma	Recurrence (%)	Liver metastasis		
Margaritora^12)^	2011	315	43 (13.7)	1	0.3	2.3
Vladislav^11)^	2012	N/A	13* (N/A)	6	N/A	N/A
Bae^13)^	2012	305	41 (13.4)	2	0.7	4.9
Sandri^14)^	2014	N/A	81 (N/A)	1	N/A	1.2
Xu^15)^	2015	331	23 (6.9)	1	0.3	4.3
Khandelwal^16)^	2016	62	14 (22.6)	3	4.8	21.4
Chiapetta^17)^	2021	N/A	135 (N/A)	7	N/A	5.2
Chiapetta^2)^	2024	1456	208 (14.3)	7	0.5	3.4

*: Number of extra thoracic recurrence.

N/A, not available

Surgery appears to be the most appropriate treatment for thymoma recurrence, offering better results compared with other types of treatments and good survival outcomes in selected patients.^2,18)^ Mizuno and Chen-Yoshikawa^3)^ reviewed the previous literature and reported recurrence rates of 9.3%–15.1% in resected thymoma cases; the proportion of patients indicated for re-resection ranged from 36.6% to 87.0%. Chiappetta et al.^2)^ reported that surgical resection ensured a favorable 5-year survival rate of over 67% in patients with distant recurrence. However, 5-year survival of patients with intra-abdominal localization is approximately 49%.

A literature review identified 19 cases of hepatic resection of metastatic thymoma to the liver. Patients with an initial histology of thymic carcinoma were excluded from this study due to significantly worse prognoses compared with thymomas. Of these 19 cases, 13 were pathologically diagnosed according to the WHO classification since 1999 and are detailed in **Table 2**.^19–31)^ Four patients had synchronous liver metastasis, and 9 had metachronous metastasis. The median recurrence-free interval was 39 months (range: 0–240 months). Solitary liver metastases were predominant, although 2 patients had multiple liver metastases. Chiappetta et al.^2)^ reported that the site of recurrence, number of localizations, and disease-free interval between primitive thymectomy and distant recurrence did not influence prognosis. However, a short disease-free survival is a risk factor for iterative recurrences.^32)^ Among the 13 patients, a second recurrence was observed in 4 patients. Of the 4 cases, 3 exhibited histological type B3, whereas the remaining case demonstrated type B1. If the pulmonary nodule in our case were a recurrence, then all cases with histological type B3 would have a second recurrence. Chiapetta et al. reported that the prognosis of patients with type B3 thymoma was significantly worse than that of patients with other thymoma subtypes. However, the prognostic role of histology in thymoma recurrence remains unclear, with controversial results reported in the literature.^2)^

**Table 2:** Clinical details of patients who underwent hepatectomy for liver metastasis of thymoma

Case	Age	Sex	Thymoma subtype		Time to liver metastasis (month)	Liver metastases		Surgery	Prognosis (months)	Second recurrence	Reference (year)
			Primary lesion	Metastatic lesion		Number	Site				
1	51	M	B3	B3	Synchronous	2	S8	Right hepatectomy	N/A	Pleural dissemination	Oikado^19)^ (2011)
2	64	F	B1	B1	42	1	S2	Left lateral sectionectomy	Alive (31)	Multiple liver metastases	Yamazaki^20)^ (2014)
3	49	F	AB	AB	57	1	Right lobe	N/A	N/A	No	Wang^21)^ (2014)
4	56	F	B1	B1	36	3	S1, S6	Wedge resection	Alive (12)	No	Nakano^22)^ (2015)
5	70	F	B2	N/A	Synchronous	1	S2	Left lateral sectionectomy	Alive (18)	No	Kimura^23)^ (2016)
6	62	M	A	A	72	1	S5/8	Right anterior sectionectomy	N/A	N/A	Kim^24)^ (2016)
7	55	F	N/A	B1	156	1	S8	Segmentectomy	Alive (6)	No	Speisky^25)^ (2016)
8	71	M	B3	B3	13	1	S4	Wedge resection	Alive (68)	Pancreatic metastasis	Passuello^26)^ (2017)
9	79	M	B1	B1	Synchronous	1	Lateral segment	Left lateral sectionectomy	Alive (42)	No	Kimura^27)^ (2018)
10	53	F	B3+B2	B3	107	1	Left lobe	Left hepatectomy	Alive (143)	Peritoneal dissemination, Liver metastasis	Nishiwaki^28)^ (2020)
11	59	F	B2	B2	240	1	S4b	Left hepatectomy	N/A	N/A	Ali^29)^ (2021)
12	56	F	B2	AB	Synchronous	1	Lateral segment	Left lateral sectionectomy	Alive (30)	No	Utsunomiya^30)^ (2021)
13	49	M	B1	B1	84	1	S5/6	Segmentectomy	N/A	N/A	Mallick^31)^ (2023)
14	63	F	B3	B3	18	1	S6	Wedge resection	Alive (16)	No	Present case

N/A, not available

Thymoma recurrence should be considered as a “chronic/long term disease”, because the recurrence rate after complete resection is very high compared with the recurrence rate after thymectomy.^32)^ Although iterative thymoma surgery has not been well investigated, complete resection of recurrent lesions is a major predictor of favorable outcome.^32,33)^ Therefore, treatment strategies for recurrent thymomas are primarily based on whether the tumor is resected upfront. Preoperative imaging plays a pivotal role in this process. The standard imaging modality for thymic tumors is intravenous contrast-enhanced CT.^33,34)^ When operative resection is planned for patients with liver metastases, the addition of preoperative MRI (either gadolinium-ethoxybenzyl-diethylenetriamine pentaacetic acid-enhanced MRI [EOB-MRI] or diffusion-weighted MRI [DW-MRI]) is recommended.^35)^ Although MRI is more sensitive than CT for detecting liver metastases, it cannot screen for metastases throughout the body.^35)^ FDG-PET/CT may reveal unexpected metastasis. According to several reports, FDG-PET/CT has been shown to detect metastatic lesions that are not visible on CT scans.^36,37)^ However, these findings were based on small-scale studies, including cases of thymic carcinoma, which tended to exhibit higher SUVmax values than thymomas. The role of FDG-PET/CT for staging purposes remains controversial,^33,34)^ primarily because of the absence of a consensus on SUV cut-offs for different stages despite the evident correlation between elevated SUV values and invasive thymic epithelial tumors.^38)^ We did not perform PET-CT before liver metastasis surgery, but it might have been worthwhile to accumulate future data.

Due to the limited data on survival after resection of liver metastases from thymoma primaries, accurate validation of treatment efficacy is challenging.^13)^ Treatment modalities for patients with liver metastasis from thymomas require extensive exploration to ascertain the optimal application of surgical options versus medical and radiological approaches.^39)^

## CONCLUSIONS

We report the case of a patient who underwent surgical resection for a solitary liver metastasis of a thymoma. Surgery remains the most appropriate treatment for solitary liver metastases; however, the optimal treatment strategy requires further elucidation due to the rarity of such cases. Therefore, accumulating a case series of patients who have undergone resection of metastatic thymoma to the liver is necessary. Identifying the clinical characteristics of patients who may benefit from surgery will aid clinicians in determining treatment indications.

## DECLARATIONS

### Availability of data and materials

The datasets supporting the conclusions of this article are included within the article.
